# Associations of harmonious and obsessive passion with attitudes toward playing through pain and injury among Chinese collegiate pickleball athletes: the mediating role of pain catastrophizing

**DOI:** 10.3389/fpsyg.2026.1871449

**Published:** 2026-06-26

**Authors:** Pengyu Cui, Ketong Jiang, Inae Yoon

**Affiliations:** 1 Department of Physical Education, Graduate School, Dankook University, Yongin-si, Gyeonggi-do, Republic of Korea; 2 Graduate School of Physical Education, Dankook University, Yongin-si, Gyeonggi-do, Republic of Korea

**Keywords:** collegiate pickleball athletes, harmonious passion, obsessive passion, pain catastrophizing, playing through pain and injury

## Abstract

**Introduction:**

This study examined the associations among harmonious passion, obsessive passion, pain catastrophizing, and attitudes toward playing through pain and injury among Chinese collegiate pickleball athletes. It was hypothesized that harmonious passion would be associated with lower pain catastrophizing and stronger opposition to playing through pain and injury, whereas obsessive passion would be associated with higher pain catastrophizing and stronger acceptance of such behavior. Pain catastrophizing was expected to be associated with weaker acceptance of playing through pain and injury and to show indirect associations linking both forms of passion with attitudes toward playing through pain and injury.

**Methods:**

Survey data were collected from 500 collegiate pickleball athletes in China. Harmonious passion and obsessive passion were assessed using the Passion Scale, pain catastrophizing was measured with the Pain Catastrophizing Scale, and attitudes toward playing through pain and injury were assessed with the Chinese version of the Risk, Pain, and Injury Questionnaire. Confirmatory factor analysis and reliability analysis supported the validity and reliability of the measures, and the hypothesized relationships were tested using structural equation modeling and bootstrap analyses.

**Results:**

The results showed that harmonious passion was associated with lower pain catastrophizing and stronger opposition to playing through pain and injury, whereas obsessive passion was associated with higher pain catastrophizing and stronger acceptance of such behavior. Contrary to the original hypothesis, pain catastrophizing was associated with stronger acceptance of playing through pain and injury. Bootstrap analyses indicated statistically significant but small indirect associations through pain catastrophizing in the relationships of both harmonious passion and obsessive passion with attitudes toward playing through pain and injury.

**Discussion:**

These findings suggest that attitudes toward playing through pain and injury among collegiate pickleball athletes may be understood in relation to passion quality and pain-related cognitive appraisal, while the indirect role of pain catastrophizing should be interpreted as modest.

## 1 Introduction

Although pickleball was initially developed as a recreational activity, it has gradually evolved into a competitive racket sport with growing global participation (Prieto-Lage et al., 2024). Although pickleball participation may provide physical, psychological, and social benefits, recent reviews have also noted emerging concerns regarding injury and musculoskeletal load as participation expands (Stroesser et al., 2024). Epidemiological evidence further suggests that pickleball-related injuries may involve overuse-related musculoskeletal problems associated with repetitive and multidirectional movement demands (Jeong et al., 2025). More importantly, continuing to participate despite pain or minor injury may not be uncommon among pickleball players (Owoeye et al., 2025). In this context, attitudes toward playing through pain and injury are relevant not only to injury management in an emerging sport, but also to athletes' psychological decision-making when pain signals arise. Such attitudes may reflect how athletes balance recovery-oriented adjustment with risk-tolerant persistence when pain or injury occurs (Wiese-Bjornstal et al., 1998; Barrette and Harman, 2020).

For Chinese collegiate pickleball athletes, this issue is also relevant. Official reports indicate the growing visibility of pickleball in Chinese university and competitive sport settings. The 2025 Chinese Collegiate Pickleball Championship reportedly involved 102 universities and more than 1,000 athletes and staff, and the National Pickleball Championship in China was organized through provincial selection and representative teams, with 25 teams competing across singles, doubles, and team events (Xinhua News Agency, 2025; General Administration of Sport of China, 2025). These reports provide contextual evidence that pickleball is becoming more visible among university athletes in China. As participation and competition opportunities expand, athletes may face training and competition demands that make attitudes toward playing through pain and injury worthy of empirical attention. Related research has shown that perceived social pressure is associated with athletes' intention to play when injured through injury-related attitudes, subjective norms, and situational temptation (Kristensen et al., 2023). Therefore, examining attitudes toward playing through pain and injury among Chinese collegiate pickleball athletes may help clarify how athletes interpret and respond to pain or injury in this emerging sport context.

Playing through pain and injury is closely tied to the psychosocial context of sport. Pain tolerance and the willingness to continue training or competition when injured or in pain are often situated within a broader culture of risk in which “playing hurt” is accepted, and are reinforced by sport-ethic beliefs such as passion for the sport, commitment, team sacrifice, minimizing pain, and acceptance of such behavior (Liston et al., 2006; Madrigal et al., 2015). Work on playing through pain and injury has also suggested that athletes' attitudes and behavioral tendencies in these situations may be shaped by athletic identity and sport culture (Weinberg et al., 2013). Empirical research has operationalized this phenomenon as attitudes and persistence tendencies in situations involving risk, pain, and injury. For example, the Risk, Pain, and Injury Questionnaire assesses attitudes and beliefs about risk, pain, and injury, including normative beliefs, and provides a basis for examining their relationships with relevant psychological variables (Walk and Wiersma, 2005). Self-Determination Theory distinguishes motivation not simply by amount, but by the quality of internalization. More autonomous forms of motivation are generally associated with better psychological and behavioral functioning, whereas more controlled forms of motivation are more likely to be linked to stress and less adaptive functioning (Ryan and Deci, 2020). Closely aligned with this perspective is the dualistic model of passion. Harmonious passion tends to reflect more autonomous internalization and flexible self-regulation, whereas obsessive passion is more likely to be accompanied by internal pressure and a more rigid pattern of persistence (Vallerand et al., 2003). A meta-analytic review of passion research has shown that harmonious and obsessive passion are differentially associated with psychological, cognitive, behavioral, and performance-related outcomes, with harmonious passion generally showing more adaptive associations and obsessive passion showing stronger links with less adaptive outcomes such as negative affect and rumination (Curran et al., 2015). In physically demanding activity contexts, passion quality has also been linked to injury-related responses. Research with dance students has shown that harmonious and obsessive passion are associated with different injury profiles and injury-related coping patterns (Rip et al., 2006). Recent work has further shown that obsessive passion is more closely related to rigid persistence, whereas harmonious passion is more closely related to flexible persistence (Chichekian and Vallerand, 2022). Accordingly, attitudes toward playing through pain and injury may differ depending on the quality of motivation, particularly because obsessive passion has been linked to rigid persistence and injury-related risky behavior in physically demanding activities (Akehurst and Oliver, 2014).

In sport-injury research, passion has been examined as a psychological factor that may influence training behavior, motivation to continue, and the suppression of fatigue and pain signals (Naderi et al., 2024). In recreational runners, higher harmonious passion strengthened the protective association between mental recovery and fewer running-related injuries, whereas obsessive passion was associated with a greater likelihood of injury (de Jonge et al., 2020). More directly, research with elite karate athletes reported that harmonious and obsessive passion were related to psychological responses to sport injury, with obsessive passion showing stronger associations with catastrophizing-related responses and harmonious passion showing greater body awareness (Ghavami et al., 2024). Related evidence from rehabilitation contexts has also linked obsessive passion for a suspended valued leisure activity with pain catastrophizing and pain intensity, with pain catastrophizing accounting for the association between obsessive passion and pain experience (Courbalay et al., 2017).

Pain catastrophizing is a negative cognitive-affective response to actual or anticipated pain, characterized by rumination, magnification, and helplessness (Sullivan et al., 1995). Research in athlete samples has shown that pain catastrophizing, particularly ruminative thinking, is associated with the subjective pain experiences of injured athletes, and that higher levels of catastrophizing are linked to reports of more severe pain (Gómez-Espejo et al., 2026). Research on athletes with musculoskeletal pain or sports-related injuries has also suggested that pain catastrophizing may help explain differences in injury management, coping, and pain-related beliefs (Maestroni et al., 2024). From a coping perspective, athletes' inclination to play through pain has been examined in relation to pain catastrophizing, ignoring pain, and reinterpreting pain sensations as pain-related coping strategies associated with physical involvement under painful conditions (Deroche et al., 2011). In addition, a recent systematic review and meta-analysis of experimental pain in athletes reported that athletes showed higher pain threshold and pain tolerance, lower pain intensity, and some evidence of differences in pain bothersomeness and performance compared with non-athletes (Thornton et al., 2024).

Pickleball-related injury research has provided an initial basis for recognizing injury concerns in this sport, but it has largely focused on injury occurrence, clinical characteristics, injury mechanisms, and healthcare utilization rather than athletes' psychological attitudes toward playing through pain and injury (Forrester, 2020; Weiss et al., 2021; Yu et al., 2025; Ahn et al., 2025; McMillan et al., 2025). In the broader sport context, qualitative syntheses of pain and injury experiences in adolescent and young adult athletes indicate that pain and injury are interpreted within social contexts that emphasize continued participation, and that the way athletes make meaning of pain and injury is central to subsequent decisions about continuation or adjustment (Sheehan et al., 2024). Therefore, direct and systematic evidence remains limited from competitive emerging racket sports, particularly among Chinese collegiate athletes, on the combined relationships among harmonious passion, obsessive passion, pain catastrophizing, and attitudes toward playing through pain and injury. Testing these associations in a collegiate athlete sample shaped by actual training and competition demands may provide preliminary evidence for understanding psychological factors related to pain-related and injury-related attitudes in emerging racket sports. Based on the above theoretical and empirical evidence, the present study examined how harmonious passion and obsessive passion are related to pain catastrophizing and attitudes toward playing through pain and injury among Chinese collegiate pickleball athletes. The study also examined whether pain catastrophizing helps explain the relationships between the two forms of passion and attitudes toward playing through pain and injury. Accordingly, the following hypotheses were proposed:

H1. Harmonious passion will be associated with stronger opposition to playing through pain and injury, whereas obsessive passion will be associated with stronger acceptance of playing through pain and injury. H2. Harmonious passion will be negatively associated with pain catastrophizing, whereas obsessive passion will be positively associated with pain catastrophizing. H3. Pain catastrophizing will be associated with weaker acceptance of playing through pain and injury. H4. Pain catastrophizing will account for a significant indirect association between harmonious passion and attitudes toward playing through pain and injury. H5. Pain catastrophizing will account for a significant indirect association between obsessive passion and attitudes toward playing through pain and injury.

## 2 Materials and methods

### 2.1 Participants

This study employed convenience sampling to recruit Chinese collegiate pickleball athletes. Questionnaires were distributed in person and collected on site with the assistance of university pickleball coaches, instructors, and student club leaders. Before completing the questionnaire, participants were informed of the study purpose, voluntary nature of participation, confidentiality of responses, and their right to withdraw. A total of 532 questionnaires were collected. After excluding 32 responses due to incomplete answers to key items, repetitive response patterns, or failure to meet the eligibility criteria, 500 valid questionnaires were retained for the final analyses.

The inclusion criteria were as follows: (1) current university enrollment in China; (2) regular participation in pickleball training for at least 6 months; (3) participation within the previous 3 months in either an official or unofficial university-, regional-, or national-level competition, or regular involvement in team training at least once per week through a club or university representative team; and (4) at least one prior experience of pickleball-related pain or injury associated with pickleball participation. Eligibility was confirmed using self-report screening items in the general demographic section of the questionnaire before the main scale items were completed.

The final sample consisted of 500 Chinese collegiate pickleball athletes, with no missing data for the demographic, sport participation, and injury-related variables. The sample included 268 male athletes (53.6%) and 232 female athletes (46.4%). Age was reported in categories: 39 participants (7.8%) were aged 18 years or younger, 161 (32.2%) were aged 19–20 years, 173 (34.6%) were aged 21–22 years, 97 (19.4%) were aged 23–24 years, and 30 (6.0%) were aged 25 years or older. Regarding grade level, 103 participants (20.6%) were first-year students, 117 (23.4%) second-year students, 105 (21.0%) third-year students, 94 (18.8%) fourth-year students, and 81 (16.2%) graduate students or above. Pickleball training experience was 6 months to 1 year for 130 participants (26.0%), 1–2 years for 166 (33.2%), 2–3 years for 126 (25.2%), and more than 3 years for 78 (15.6%). Self-rated pickleball level was below 3.0 for 117 participants (23.4%), 3.1–4.0 for 221 (44.2%), 4.1–5.0 for 139 (27.8%), and 5.1 or above for 23 (4.6%). Weekly training frequency was once per week for 96 participants (19.2%), 2–3 times per week for 225 (45.0%), 4–5 times per week for 134 (26.8%), and 6 times or more per week for 45 (9.0%). The main reported sites of pickleball-related pain or injury were the knee (*n* = 97, 19.4%), ankle/ foot (*n* = 86, 17.2%), lower back/back (*n* = 75, 15.0%), shoulder (*n* = 72, 14.4%), wrist/ hand (*n* = 69, 13.8%), elbow (*n* = 57, 11.4%), hip (*n* = 31, 6.2%), and other sites (*n* = 13, 2.6%). The most recent episode of pickleball-related pain or injury occurred within 1 month for 125 participants (25.0%), 1–3 months ago for 148 (29.6%), 3–6 months ago for 148 (29.6%), and more than 6 months ago for 79 (15.8%). Regarding self-reported severity of the most recent pickleball-related pain or injury episode, 204 participants (40.8%) reported mild symptoms, 187 (37.4%) reported moderate symptoms, 88 (17.6%) reported relatively severe symptoms, and 21 (4.2%) reported severe symptoms.

### 2.2 Measures

#### 2.2.1 Passion

Passion was assessed using the Passion Scale developed by Vallerand et al. (2003). The Chinese version validated by Zhao et al. (2015) was used in the present study. In that validation study, CFA supported the two-factor structure with acceptable model fit (CFI = 0.939, TLI = 0.921, RMSEA = 0.069), and the internal consistency coefficients were.86 for harmonious passion and.82 for obsessive passion. In the present study, Cronbach's α values were.867 for harmonious passion, 0.894 for obsessive passion, and.832 for the overall scale. The scale consists of 12 items contextualized to pickleball training and competition across two subscales: harmonious passion (6 items; e.g., “My pickleball training and competition are in harmony with the other activities in my life”) and obsessive passion (6 items; e.g., “I have difficulty controlling my urge to participate in pickleball training and competition”). Responses were recorded on a 7-point Likert scale ranging from 1 (not at all) to 7 (very strongly).

#### 2.2.2 Pain catastrophizing

Pain catastrophizing was measured using the Pain Catastrophizing Scale developed by Sullivan et al. (1995). The simplified Chinese version validated by Xu et al. (2015) was used in this study. In that validation study, CFA supported a second-order three-factor structure with acceptable model fit (CMIN/DF = 1.681, NNFI = 0.948, CFI = 0.96, GFI = 0.907, RMSEA = 0.068), and Cronbach's α values were.91 for the total scale and.85–0.88 for the three subscales. In the present study, Cronbach's α values were.822 for rumination, 0.744 for magnification, 0.860 for helplessness, and 0.907 for the overall scale. The scale consists of 13 items across three subscales: rumination (4 items; e.g., “I cannot get the thought of pain out of my mind”), magnification (3 items; e.g., “I worry that something bad may happen”), and helplessness (6 items; e.g., “There is nothing I can do to reduce the pain”). Each item was rated on a 5-point scale ranging from 0 to 4.

#### 2.2.3 Attitudes toward playing through pain and injury

Attitudes toward playing through pain and injury were assessed using the Chinese version of the Risk, Pain, and Injury Questionnaire. The original Risk, Pain, and Injury Questionnaire was reexamined for construct validity by Walk and Wiersma (2005), and the Chinese version for collegiate athletes was validated by Liu et al. (2023). In that validation study, CFA supported the 12-item three-factor structure with acceptable model fit (CMIN/DF = 2.61, CFI = 0.93, TLI = 0.91, RMSEA = 0.07), and Cronbach's α values were.70 for tough, 0.69 for pressed, and.76 for rational choice. In the present study, Cronbach's α values were.769 for tough, 0.836 for pressed, 0.864 for rational choice, and.886 for the overall scale. The scale includes 12 items across three subscales: tough (3 items; e.g., “Athletes should ignore pain”), pressed (4 items; e.g., “Coaches are impressed with athletes who play with injuries and pain”), and rational choice (5 items; e.g., “Athletes who endure pain and play hurt deserve our respect”). Responses were recorded on a 4-point Likert scale ranging from 1 (strongly agree) to 4 (strongly disagree). Higher scores indicate stronger disagreement with statements endorsing continued sport participation despite pain or injury, whereas lower scores indicate stronger agreement with such statements.

### 2.3 Validity and reliability of the measures

To examine the validity and reliability of the instruments used in this study, confirmatory factor analysis was conducted. Model fit was evaluated using the following criteria: Tucker-Lewis index values greater than 0.90, comparative fit index values greater than 0.90, and root mean square error of approximation values below 0.08. The results of the validity and reliability analyses for harmonious passion, obsessive passion, pain catastrophizing, and attitudes toward playing through pain and injury are presented in Table 1.

**Table 1:** Validity and reliability of the measures.

Factor		Item	*B*	*S.E*.	*C.R*.	β	AVE	CR
Passion	Harmonious passion	1	1			0.684	0.526	0.868
		3	1.124	0.074	15.284^***^	0.781		
		5	0.865	0.070	12.276^***^	0.610		
		6	0.967	0.072	13.351^***^	0.669		
		8	1.130	0.073	15.461^***^	0.793		
		10	1.143	0.074	15.469^***^	0.793		
	Obsessive passion	2	1			0.846	0.589	0.895
		4	0.954	0.047	20.369^***^	0.789		
		7	0.815	0.049	16.670^***^	0.682		
		9	0.883	0.047	18.834^***^	0.746		
		11	0.891	0.046	19.422^***^	0.763		
		12	0.933	0.048	19.608^***^	0.768		
	Model fit indices	χ^2^ = 135.635, *df* = 53 χ^2^/df = 2.559, GFI = 0.957, CFI = 0.971, TLI = 0.964, RMSEA = 0.056, SRMR = 0.060						
Pain catastrophizing	Rumination	8	1			0.774	0.546	0.826
		9	1.032	0.059	17.629^***^	0.801		
		10	1.031	0.060	17.263^***^	0.784		
		11	0.732	0.059	12.349^***^	0.573		
	Magnification	6	1			0.752	0.535	0.775
		7	0.878	0.065	13.482^***^	0.688		
		13	0.978	0.068	14.392^***^	0.753		
	Helplessness	1	1			0.785	0.582	0.893
		2	0.951	0.055	17.260^***^	0.739		
		3	0.930	0.056	16.737^***^	0.720		
		4	1.059	0.054	19.621^***^	0.822		
		5	0.948	0.055	17.187^***^	0.736		
		12	1.001	0.055	18.121^***^	0.769		
	Model fit indices	χ^2^ = 106.730, *df* = 62, χ^2^/df = 1.721, GFI = 0.968, CFI = 0.986, TLI = 0.982, RMSEA = 0.038, SRMR = 0.031						
Attitudes toward playing through pain and injury	Tough	1	1			0.729	0.526	0.769
		2	1.023	0.075	13.565^***^	0.762		
		3	0.931	0.073	12.801^***^	0.685		
	Pressed	4	1			0.783	0.562	0.837
		5	0.942	0.056	16.767^***^	0.769		
		6	0.958	0.058	16.533^***^	0.758		
		7	0.860	0.058	14.894^***^	0.686		
	Rational choice	8	1			0.657	0.574	0.871
		9	1.282	0.081	15.808^***^	0.861		
		10	1.138	0.079	14.436^***^	0.759		
		11	1.092	0.079	13.788^***^	0.717		
		12	1.123	0.079	14.238^***^	0.746		
	Model fit indices	χ^2^ = 87.941, *df* = 51, χ^2^/df = 1.724, GFI = 0.971, CFI = 0.986, TLI = 0.982, RMSEA = 0.038, SRMR = 0.030						

AVE, average variance extracted; CR, composite reliability. ^***^p <0.001.

### 2.4 Control and assessment of common method bias

To control for and assess common method bias, confirmatory factor analysis and reliability testing were first conducted for the measurement instruments. In addition, Harman's single-factor test was performed to assess common method bias (Podsakoff et al., 2003). The unrotated principal component analysis extracted eight factors with eigenvalues greater than 1, and the first factor accounted for 26.544% of the total variance, which was below the 40% threshold. These results suggest that common method bias was not a serious concern in the present study. As a supplementary assessment, full collinearity VIFs were calculated for the main constructs. The VIF values ranged from 1.178 to 1.422, which were below the commonly used threshold of 3.3 (Kock, 2015). Thus, the VIF results did not indicate substantial full collinearity or serious concern regarding common method bias.

### 2.5 Data analysis

All data analyses were performed using SPSS 27.0 and AMOS 28.0. After questionnaire screening, the final dataset contained no missing values for the variables used in the analyses; therefore, no missing-value imputation was performed. Frequency analyses were conducted for participant characteristics, and descriptive statistics, including means, standard deviations, skewness, and kurtosis, were calculated for the main study variables and their subdimensions. Confirmatory factor analysis and reliability analysis were first conducted to evaluate the validity and reliability of the study measures. Harman's single-factor test and full collinearity VIFs were then used to assess common method bias. Pearson correlation analysis was performed to examine the relationships among the study variables and their subdimensions. An overall measurement model was examined before testing the structural relationships. Structural equation modeling was then conducted to test the hypothesized relationships among harmonious passion, obsessive passion, pain catastrophizing, and attitudes toward playing through pain and injury. The indirect effects were examined using bias-corrected bootstrap analysis with 5,000 resamples and 95% confidence intervals. An additional covariate-adjusted structural model was specified by entering self-rated pickleball level, injury recency, and self-reported injury severity as observed covariates predicting pain catastrophizing and attitudes toward playing through pain and injury. Statistical significance was set at *p* < 0.05.

### 2.6 Ethics statement

The studies involving human participants were reviewed and approved by the Baoshan University Ethics Committee, Baoshan University, China. The study was conducted in accordance with the Declaration of Helsinki, institutional ethical guidelines, and the relevant laws and regulations of the People's Republic of China. Written informed consent was obtained from all participants prior to participation.

## 3 Results

### 3.1 Correlations among the variables

Correlation analysis was conducted to examine the relationships among the study variables. The results are presented in Table 2. Harmonious passion and obsessive passion showed statistically significant correlations with the subdimensions of pain catastrophizing, namely rumination, magnification, and helplessness, as well as with the subdimensions of attitudes toward playing through pain and injury, namely tough, pressed, and rational choice. Specifically, harmonious passion was negatively correlated with rumination, magnification, and helplessness (all *ps* < 0.01), and positively correlated with tough, pressed, and rational choice (all *ps* < 0.01). In contrast, obsessive passion was positively correlated with rumination, magnification, and helplessness (all *ps* < 0.01), and negatively correlated with tough, pressed, and rational choice (all *ps* < 0.01).

**Table 2:** Correlations among the variables.

Variable	1	2	3	4	5	6	7	8
1.Harmonious passion	1							
2. Obsessive passion	0.121^**^	1						
3.Rumination	−0.144^**^	0.369^**^	1					
4.Magnification	−0.210^**^	0.269^**^	0.546^**^	1				
5.Helplessness	−0.179^**^	0.354^**^	0.596^**^	0.507^**^	1			
6.Tough	0.216^**^	−0.329^**^	−0.297^**^	−0.248^**^	−0.341^**^	1		
7.Pressed	0.211^**^	−0.257^**^	−0.282^**^	−0.234^**^	−0.345^**^	0.439^**^	1	
8.Rational choice	0.218^**^	−0.281^**^	−0.316^**^	−0.269^**^	−0.368^**^	0.480^**^	0.531^**^	1
*M*	4.005	3.980	2.003	1.981	1.983	2.516	2.517	2.493
*SD*	1.547	1.610	1.159	1.182	1.146	0.916	0.908	0.906
Skewness	0.008	0.079	0.004	0.121	0.062	−0.019	0.005	0.038
Kurtosis	−0.909	−1.013	−1.098	−1.121	−1.047	−1.090	−1.120	−1.097

Skewness and kurtosis values were within acceptable ranges. ^**^p < 0.01.

### 3.2 Measurement model

Following the two-step approach proposed by Anderson and Gerbing (1988), the measurement model was first examined through confirmatory factor analysis before testing the structural relationships and mediating effects. As shown in Table 3, the measurement model demonstrated a good fit to the data, with χ^2^ = 848.917, *df* = 601, χ^2^/ *df* = 1.413, GFI = 0.917, CFI = 0.972, TLI = 0.969, RMSEA = 0.029, and SRMR = 0.040. These results support the adequacy of the measurement model.

**Table 3:** Measurement model results.

Latent variable	Item	*B*	*S.E*.	*C.R*.	β
Harmonious passion	A1	1			0.687
	A3	1.116	0.073	15.375^***^	0.779
	A5	0.865	0.070	12.395^***^	0.613
	A6	0.963	0.072	13.450^***^	0.670
	A8	1.127	0.072	15.617^***^	0.794
	A10	1.133	0.073	15.539^***^	0.789
Obsessive passion	A2	1			0.843
	A4	0.959	0.047	20.411^***^	0.790
	A7	0.821	0.049	16.741^***^	0.684
	A9	0.888	0.047	18.885^***^	0.747
	A11	0.895	0.046	19.433^***^	0.763
	A12	0.938	0.048	19.665^***^	0.769
Rumination	B8	1			0.772
	B9	1.035	0.059	17.663^***^	0.802
	B10	1.033	0.060	17.267^***^	0.783
	B11	0.737	0.059	12.411^***^	0.575
Magnification	B6	1			0.750
	B7	0.877	0.065	13.492^***^	0.685
	B13	0.986	0.068	14.527^***^	0.757
Helplessness	B1	1			0.787
	B2	0.947	0.055	17.330^***^	0.738
	B3	0.931	0.055	16.893^***^	0.723
	B4	1.055	0.053	19.741^***^	0.821
	B5	0.946	0.055	17.309^***^	0.737
	B12	0.993	0.055	18.101^***^	0.765
Tough	C1	1			0.734
	C2	0.996	0.072	13.794^***^	0.748
	C3	0.937	0.071	13.159^***^	0.695
Pressed	C4	1			0.783
	C5	0.944	0.056	16.842^***^	0.770
	C6	0.960	0.058	16.594^***^	0.759
	C7	0.857	0.058	14.875^***^	0.683
Rational choice	C8	1			0.657
	C9	1.279	0.081	15.832^***^	0.860
	C10	1.139	0.079	14.477^***^	0.760
	C11	1.091	0.079	13.803^***^	0.717
	C12	1.124	0.079	14.270^***^	0.747
Model fit indices	χ^2^ = 848.917, *df* = 601, χ^2^/df = 1.413, GFI = 0.917, CFI = 0.972, TLI = 0.969, RMSEA = 0.029, SRMR = 0.040				

^***^p < 0.001.

### 3.3 Structural model

To test the mediating role of pain catastrophizing in the associations of harmonious passion and obsessive passion with attitudes toward playing through pain and injury, a structural model was specified. Harmonious passion and obsessive passion were modeled as exogenous variables, whereas pain catastrophizing and attitudes toward playing through pain and injury were modeled as endogenous variables. Paths from harmonious passion and obsessive passion to pain catastrophizing were specified to examine the hypothesized associations between the two forms of passion and pain-related cognitive and emotional responses. A path from pain catastrophizing to attitudes toward playing through pain and injury was specified to examine the hypothesized association between pain-related cognitive appraisal and attitudes toward continued sport participation under painful or injured conditions. Direct paths from harmonious passion and obsessive passion to attitudes toward playing through pain and injury were also included.

**Figure 1:**
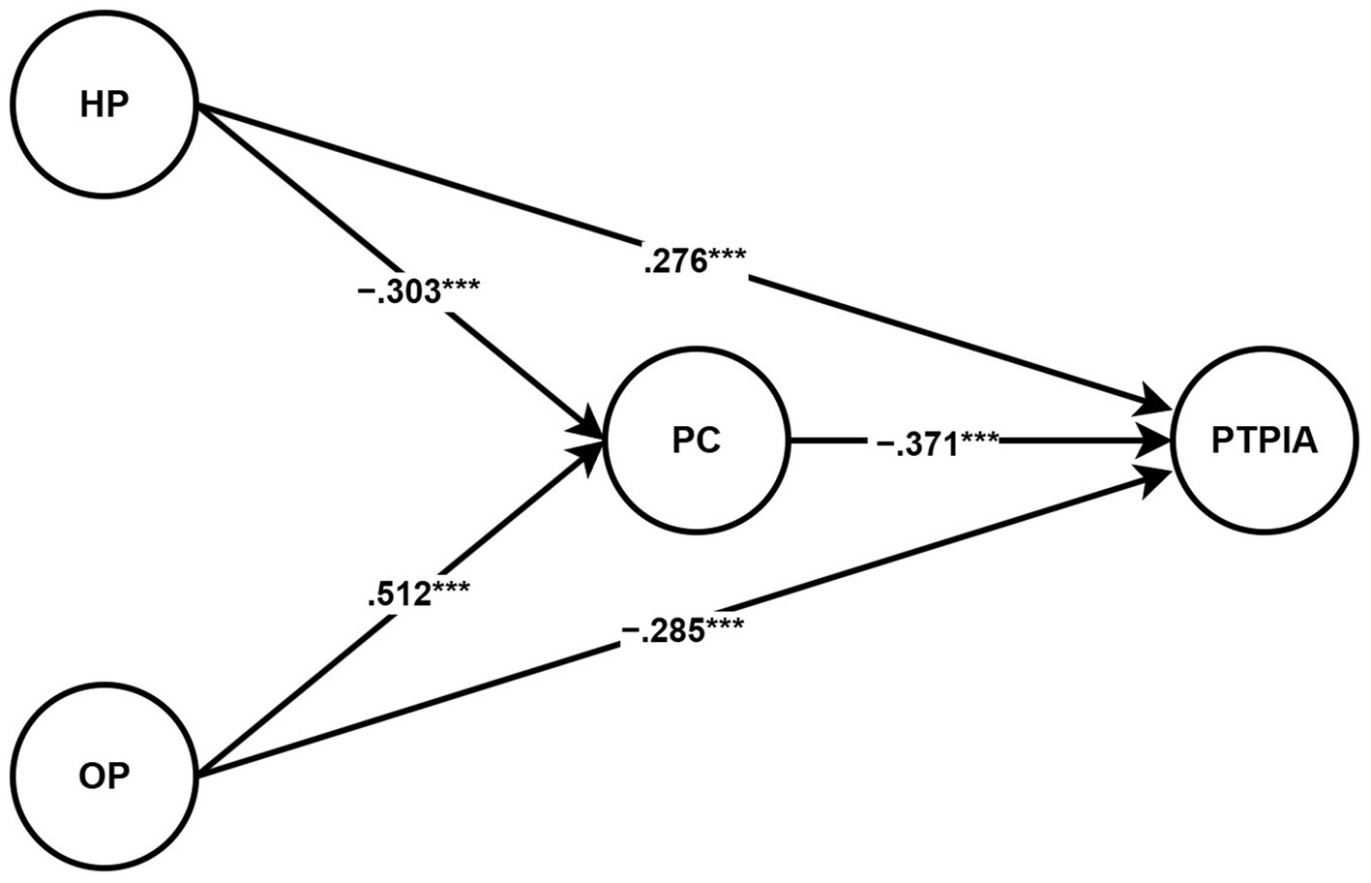
Structural model of the associations among harmonious passion, obsessive passion, pain catastrophizing, and attitudes toward playing through pain and injury. Standardized path coefficients are shown. ***p < 0.001. Abbreviations: HP, harmonious passion; OP, obsessive passion; PC, pain catastrophizing; PTPIA, attitudes toward playing through pain and injury.

The structural model showed a good fit to the data, with χ^2^ = 872.544, df = 617, χ^2^/ df = 1.414, GFI = 0.915, CFI = 0.971, TLI = 0.969, RMSEA = 0.029, and SRMR = 0.043. Harmonious passion showed a significant negative direct path to pain catastrophizing (β = −0.303, *p* < 0.001), whereas obsessive passion showed a significant positive direct path to pain catastrophizing (β = 0.512, *p* < 0.001). Pain catastrophizing showed a significant negative direct path to attitudes toward playing through pain and injury (β = −0.371, *p* < 0.001). In addition, harmonious passion showed a significant positive direct path to attitudes toward playing through pain and injury (β = 0.276, *p* < 0.001), whereas obsessive passion showed a significant negative direct path (β = −0.285, *p* < 0.001). The results of the structural model are presented in Table 4 and Figure 1.

**Table 4:** Structural model results.

Path	*B*	β	*S.E*.	*t*
Harmonious passion pain catastrophizing	−0.182	−0.303	0.031	−5.919^***^
Obsessive passion → pain catastrophizing	0.312	0.512	0.035	9.012^***^
Pain catastrophizing → attitudes toward playing through pain and injury	−0.240	−0.371	0.046	−5.208^***^
Harmonious passion → attitudes toward playing through pain and injury	0.107	0.276	0.022	4.908^***^
Obsessive passion → attitudes toward playing through pain and injury	−0.112	−0.285	0.025	−4.536^***^
Model fit indices	χ^2^ = 872.544, *df* = 617, χ^2^/df = 1.414, GFI = 0.915, CFI = 0.971, TLI = 0.969, RMSEA = 0.029, SRMR = 0.043			

^***^p < 0.001.

Given the scoring direction of the Risk, Pain, and Injury Questionnaire, these results supported H1 and H2. However, pain catastrophizing was associated with stronger acceptance of playing through pain and injury, which was opposite to H3; therefore, H3 was not supported.

### 3.4 Mediation analysis

Bootstrap analysis was conducted to determine whether the indirect effects through pain catastrophizing were statistically significant (Shrout and Bolger, 2002). Bias-corrected estimation was applied, with 5,000 bootstrap samples and 95% confidence intervals. The indirect effect of harmonious passion on attitudes toward playing through pain and injury through pain catastrophizing was statistically significant but small in magnitude [*B* = 0.044, 95% CI (0.024, 0.068), *p* < 0.001]. The indirect effect of obsessive passion on attitudes toward playing through pain and injury through pain catastrophizing was also statistically significant but small in magnitude [*B* = −0.075, 95% CI (−0.107, −0.045), *p* < 0.001]. Because the direct effects of harmonious passion and obsessive passion on attitudes toward playing through pain and injury also remained significant [β = 0.276, *p* < 0.001; β = −0.285, *p* < 0.001, respectively], pain catastrophizing was interpreted as a modest partial mediator in both relationships. The mediation results are presented in Table 5.

**Table 5:** Mediation results.

Path	*B*	95% CI (L, U)	*p*
Harmonious passion → pain catastrophizing → attitudes toward playing through pain and injury	0.044	[0.024, 0.068]	<0.001
Obsessive passion → pain catastrophizing → attitudes toward playing through pain and injury	−0.075	[−0.107, −0.045]	<0.001

Bootstrap = 5,000 samples; 95% CI = bias-corrected percentile method.

These mediation results supported H4 and H5, indicating that pain catastrophizing showed significant indirect associations linking both forms of passion with attitudes toward playing through pain and injury.

### 3.5 Covariate-adjusted structural model

An additional covariate-adjusted structural model was tested by including self-rated pickleball level, injury recency, and self-reported severity of the most recent pain or injury episode as covariates. The model showed an acceptable fit to the data: χ^2^ = 986.662, *df* = 716, χ^2^/ *df* = 1.378, GFI = 0.912, CFI = 0.970, TLI = 0.967, and RMSEA = 0.028. The main structural paths remained significant. Harmonious passion showed a significant negative path to pain catastrophizing (β = −0.300, *p* < 0.001), whereas obsessive passion showed a significant positive path to pain catastrophizing (β = 0.490, *p* < 0.001). Pain catastrophizing showed a significant negative path to attitudes toward playing through pain and injury (β = −0.384, *p* < 0.001). Harmonious passion showed a significant positive path to attitudes toward playing through pain and injury (β = 0.271, *p* < 0.001), whereas obsessive passion showed a significant negative path to attitudes toward playing through pain and injury (β = −0.286, *p* < 0.001). Among the covariates, self-reported injury severity showed a significant positive path to pain catastrophizing (β =0.172, p <0.001), whereas self-rated pickleball level and injury recency did not show significant paths to pain catastrophizing. None of the covariates showed significant paths to attitudes toward playing through pain and injury. Overall, the covariate-adjusted results were consistent with the main analyses. H1, H2, H4, and H5 remained supported, whereas H3 remained unsupported.

## 4 Discussion

This study focused on Chinese collegiate pickleball athletes and examined the structural relationships among harmonious passion, obsessive passion, pain catastrophizing, and attitudes toward playing through pain and injury. Drawing on self-determination theory, the dualistic model of passion, and pain-related cognitive appraisal perspectives, the results showed that harmonious passion and obsessive passion were associated with attitudes toward playing through pain and injury in opposite directions and were also differentially associated with pain catastrophizing. Specifically, H1 and H2 were supported, whereas H3 was not supported because pain catastrophizing was associated with stronger, rather than weaker, acceptance of playing through pain and injury. H4 and H5 were supported, although the indirect associations through pain catastrophizing were small in magnitude. These findings suggest that attitudes toward playing through pain and injury among collegiate pickleball athletes may be shaped by the quality of passion and by how athletes cognitively appraise pain.

### 4.1 Associations between passion and attitudes toward playing through pain and injury (H1 supported)

Consistent with H1, harmonious passion was associated with stronger opposition to playing through pain and injury, whereas obsessive passion was associated with stronger acceptance of such behavior. Harmonious passion may therefore be better understood not as simple toughness, but as a more flexible and self-regulated form of persistence that may support more adaptive appraisal of pain and injury risk (Rip et al., 2006; Vallerand et al., 2023). From this perspective, harmonious passion may reflect a greater willingness to use pain signals as information and to distance oneself from unnecessary risk. Obsessive passion, in contrast, may reflect a motivational orientation in which internal pressure to remain involved is accompanied by greater acceptance of sport-ethic beliefs that normalize continued participation despite pain or injury. This interpretation is consistent with evidence linking obsessive passion to dependence-like involvement, rigid persistence, and injury-related risky behavior, as well as with research showing that playing through pain and injury is embedded in communicated norms, perceived social pressure, and overconformity to the sport ethic (Akehurst and Oliver, 2014; Capra and LaBelle, 2022; Coker-Cranney et al., 2018; Kristensen et al., 2023). Walk and Wiersma (2005) argued that the attitudes measured by the Risk, Pain, and Injury Questionnaire should not be understood as simple dispositions, but as structured attitudes shaped by both cultural and cognitive factors. The present findings are consistent with that view.

### 4.2 Associations between passion and pain catastrophizing (H2 supported)

Consistent with H2, harmonious passion was negatively associated with pain catastrophizing, whereas obsessive passion was positively associated with pain catastrophizing. This pattern suggests that the two forms of passion may be differentially associated with athletes' pain-related cognitive appraisal, rather than simply reflecting the degree of sport involvement (Lavoie et al., 2021; Sullivan et al., 2000). Sport-specific evidence provides a closer basis for interpreting this pattern. Among college athletes, pain catastrophizing has been associated with injury status and pain, with athletes reporting current or previous injury, or playing with pain, showing higher catastrophizing than those without injury or pain (Sciascia et al., 2020). In competitive runners, obsessive passion has been identified as a positive predictor of perceived susceptibility to sport-related injury, whereas harmonious passion showed a negative relation in separate analyses (Stephan et al., 2009). In addition, research linking the two dimensions of passion to excessive and dependent exercise behavior indirectly supports the closer affinity between obsessive passion and rigid forms of persistence (Szabo et al., 2022). Thus, the positive association between obsessive passion and pain catastrophizing observed in the present study may indicate stronger cognitive-emotional responses such as rumination, magnification, and helplessness, thereby making continued participation more likely to be justified even under painful conditions.

### 4.3 Association between pain catastrophizing and attitudes toward playing through pain and injury (H3 not supported)

Contrary to H3, pain catastrophizing was associated with stronger acceptance of playing through pain and injury. This finding was unexpected because pain catastrophizing is commonly discussed within fear-avoidance perspectives, in which catastrophic interpretations of pain are linked to pain-related fear, avoidance behavior, disability, and poorer physical functioning (Vlaeyen and Linton, 2000; Leeuw et al., 2007; Fischerauer et al., 2018). From this perspective, athletes with higher pain catastrophizing might be expected to show less acceptance of continued participation under pain or injury conditions. However, pain in sport is often experienced within training and competition contexts where withdrawal is not determined by pain appraisal alone. Research comparing athletes and non-athletes with low back pain has shown that endurance responses can be more frequent than avoidance responses, suggesting that pain-related threat does not necessarily lead to withdrawal in athlete populations (Gajsar et al., 2019). Qualitative studies further show that playing through pain and injury is often embedded in sport cultures in which athletes may minimize pain, prioritize team contribution, accept sacrifice, and regard playing hurt as a normal or expected part of participation (Liston et al., 2006; Madrigal et al., 2015; Barrette and Harman, 2020). Social and interpersonal pressures may also help explain why higher pain catastrophizing can coexist with stronger acceptance of continued participation. Studies on pain and injury management have shown that coaches, teammates, medical staff, parents, and spectators can shape athletes' decisions to disclose symptoms, seek help, or continue participation despite injury (Nixon, 1994; Roderick et al., 2000; Kroshus et al., 2015). In youth sport, many athletes report hiding injuries to continue playing, and both players and coaches report witnessing pressure to play when injured (Whatman et al., 2018). Therefore, in the present collegiate pickleball context, higher pain catastrophizing may reflect a heightened perception of pain-related threat while also coexisting with sport-specific norms and social expectations that make continued participation under pain or injury more acceptable.

### 4.4 Indirect associations through pain catastrophizing (H4 and H5 supported)

Consistent with H4 and H5, pain catastrophizing showed statistically significant but small indirect associations in the relationships of harmonious passion and obsessive passion with attitudes toward playing through pain and injury. Because the direct associations also remained significant, pain catastrophizing can be interpreted as a modest partial mediator in both relationships. This interpretation is meaningful because pain catastrophizing represents a cognitive-affective response to pain, including rumination, magnification, and helplessness (Sullivan et al., 1995). In sport contexts, pain catastrophizing has been associated with athletes' pain intensity, pain unpleasantness, and perceived pain severity during training, competition, or injury-related situations (Gagnon-Dolbec et al., 2021; Gómez-Espejo et al., 2026). Therefore, in the present study, pain catastrophizing may be understood as a modest pain-related cognitive-affective correlate involved in the indirect associations between passion quality and attitudes toward continued participation under pain or injury conditions.

### 4.5 Practical implications

The present findings may be useful for pain- and injury-related communication with collegiate pickleball athletes. In coaching and training settings, messages that simply encourage athletes to “endure” or “keep going” may not be appropriate for all athletes. The results suggest that coaches, instructors, and sport support staff should pay attention to differences in passion quality and pain catastrophizing when discussing whether athletes should continue participation under pain or injury conditions. Athletes with higher obsessive passion and higher pain catastrophizing may need closer attention, because this pattern was associated with stronger acceptance of playing through pain and injury in the present study. For these athletes, communication may need to emphasize careful interpretation of pain signals and the importance of avoiding unnecessary injury risk. In contrast, athletes with higher harmonious passion may be more likely to respond to messages that support balanced risk appraisal and self-regulated participation. Overall, pain- and injury-related communication in collegiate pickleball should not simply praise persistence, but should help athletes make more careful judgments about continued participation when pain or injury occurs.

## 5 Conclusion

This study examined the associations among harmonious passion, obsessive passion, pain catastrophizing, and attitudes toward playing through pain and injury among Chinese collegiate pickleball athletes. Harmonious passion was associated with lower pain catastrophizing and stronger opposition to playing through pain and injury, whereas obsessive passion was associated with higher pain catastrophizing and stronger acceptance of such behavior. Pain catastrophizing was also associated with stronger acceptance of playing through pain and injury, contrary to the original hypothesis. In addition, pain catastrophizing showed statistically significant but small partial mediating effects in the associations of both forms of passion with attitudes toward playing through pain and injury. These findings suggest that attitudes toward playing through pain and injury in collegiate pickleball may be understood in relation to both passion quality and pain-related cognitive appraisal, although the mediating role of pain catastrophizing should be interpreted as modest.

Several limitations should be noted. First, although the covariate-adjusted model included self-rated pickleball level, injury recency, and self-reported injury severity, other relevant factors such as current pain intensity, injury frequency, clinically verified injury diagnosis, training load, coaching climate, and perceived social pressure were not assessed. Future studies should include more detailed injury-related, training-related, and social-contextual variables. Second, the cross-sectional design does not allow conclusions about temporal order or causality. Reverse causality and third-variable confounding cannot be ruled out; for example, athletes' attitudes toward playing through pain and injury may also shape how they appraise pain, and unmeasured sport-contextual factors may influence both pain catastrophizing and such attitudes. Longitudinal, prospective, or intervention-based studies are needed to clarify causal pathways. Third, the sample was limited to Chinese collegiate pickleball athletes, which may restrict the generalizability of the findings. Future research should examine athletes from different sports, competitive levels, age groups, and cultural contexts.

## Data Availability

The raw data supporting the conclusions of this article will be made available by the authors, without undue reservation.
